# Use of a Modified Nance Appliance for Esthetic Rehabilitation of a Child Patient with Rare Nonfamilial and Nonsyndromic Oligodontia

**DOI:** 10.1155/2021/5444257

**Published:** 2021-07-21

**Authors:** Abbas O. Al-Ahmadi, Ali A. Assiry, Siraj D. A. A. Khan, Anand Marya, Adith Venugopal, Mohmed Isaqali Karobari

**Affiliations:** ^1^Specialized Dental Centre, King Fahad Hospital, Medina, Saudi Arabia; ^2^Pediatric Dentistry, Faculty of Dentistry, Najran University, Saudi Arabia; ^3^Department of Orthodontics, Faculty of Dentistry, University of Puthisastra, Phnom Penh, Cambodia; ^4^Department of Orthodontics, Saveetha Dental College, Saveetha Institute of Medical and Technical Sciences, Saveetha University, Chennai, India; ^5^Conservative Unit, School of Dental Sciences, Universiti Sains Malaysia, Health Campus, Kubang Kerian, 16150 Kota Bharu, Kelantan, Malaysia

## Abstract

Congenital absence of more than 6 teeth except the third molar is known as oligodontia. It can affect both primary and permanent dentitions. The affected individuals usually demonstrate esthetic, functional, and psychological concerns. The present case report is about a 4-year-old female patient with multiple missing (both primary and permanent) teeth with esthetic and social problems. On extraoral examination, the patient demonstrated upper lip relapse due to missing anterior teeth. On physical examination, no other abnormality was detected in relation to the hair, appendages, or presence of a cleft. On intraoral and radiographic examination, 21 teeth were missing, which included both the primary and the permanent tooth buds. To encourage a positive behavioral change, it was decided to carry out prosthetic intervention using a modified fixed Nance appliance and acrylic teeth. After the replacement of the missing teeth, a marked improvement in the profile and esthetics was seen.

## 1. Introduction

The tooth is one of the most useful tissues in the body. Dentinogenesis is a dynamic process resulting from many instructive and permissive cellular and molecular interactions; any disturbance across these interactions will affect tooth development either partly or entirely [1, 2].

Congenital absence of one or more teeth either in primary or permanent dentition is known as tooth agenesis, which could be partial (hypodontia or oligodontia) or complete (anodontia), excluding the third molar [3, 4]. Oligodontia is a rare condition that can occur either in association with genetic syndromes, as a nonsyndromic isolated familial trait or sporadic finding [5]. It mainly occurs in the permanent dentition but can affect primary dentition also [6]. In the primary dentition, it is seen in the incisal region and is often associated with missing succedaneous teeth [7].

In young patients, the absence of teeth can cause esthetic, functional, and psychological problems, especially when the anterior teeth are involved. This paper presents a unique case of a young child with nonfamilial nonsyndromic oligodontia, present in both primary and permanent dentition, where esthetic, form, and functions of dentition were established through prosthetic intervention.

## 2. Case Report

A 4-year-old female patient reported to the Department of Pediatric Dentistry at King Fahad Hospital with her father. Her father stated that she demonstrated negative behavioral changes due to multiple missing teeth and avoided social activities. The patient was apprehensive and depicting a negative attitude during a routine dental examination. No significant familial history regarding the absence of teeth was reported. The dental history given by the parents suggested that there was a congenital absence of most of the primary teeth.

**Figure 1:**
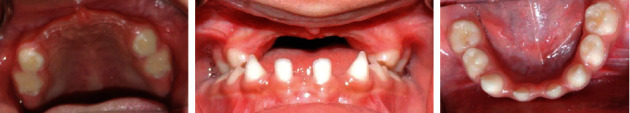
Intraoral pictures demonstrating multiple missing teeth.

**Figure 2:**
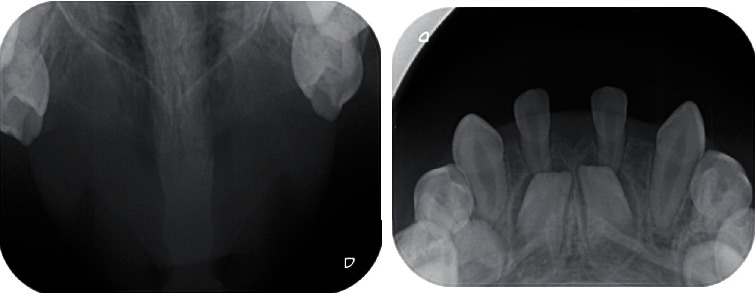
Occlusal view of both the maxillary and the mandibular arches.

Intraoral examination during the first visit revealed that the dentition was spaced, there is absence of 8 primary teeth (tooth numbers 51, 52, 53, 61, 62, 63, 72, and 82), and both right and left lower canines were conical in shape (Figure 1). The alveolus of the upper anterior was very thin, and the teeth present were of standard size, shape, and color. The occlusal view of both maxillary and mandibular (Figure 2) arches revealed the absence of 13 tooth buds (tooth numbers 11, 12, 13, 15, 21, 22, 23, 25, 32, 33, 35, 42, and 45). Provisionally, it was diagnosed as nonfamilial and nonsyndromic oligodontia.

To rule out the syndromic and genetic involvement, the patient was referred to a pediatrician and a genetic consultant, both of whom confirmed that it was a case of nonfamilial and nonsyndromic oligodontia.

## 3. Treatment Plan

**Figure 3:**
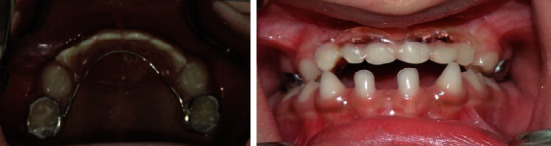
Modified fixed Nance appliance with artificial anterior teeth.

**Figure 4:**
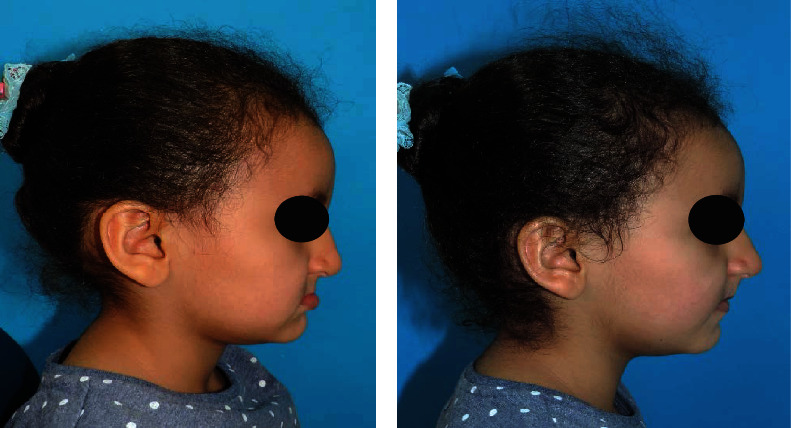
Pretreatment and postplacement of the appliance showing definite improvement in the patient's profile.

A modified fixed Nance appliance with artificial anterior teeth (Figure 3) was planned for esthetic rehabilitation along with careful behavior management. Since the patient's chief concern was esthetics, it was decided only to rehabilitate the maxillary arch that would help improve her nasolabial profile. This modified appliance was cemented on tooth numbers 55 and 65. With improvement in esthetics, the patient immediately reported a positive change in attitude (Figure 4). After one week, during the follow-up visit, the patient's parents reported a marked improvement in her social interactions and positive behavior.

## 4. Discussion

The biological basis of tooth agenesis is a sequential and reciprocal series of signaling in a precisely organized manner that leads to the lingual and distal proliferation of the tooth germ from the dental lamina. Any disturbance during this process will affect tooth development either partly or entirely [8, 9].

Various etiological factors have been proposed for tooth agenesis which varies from environmental factors such as radiotherapy, tumors, trauma to the alveolar processes, disturbance in the innervation of the jaw, hormonal influences, rubella, and drugs like thalidomide to hereditary factors such as MSX1 and PAX9, AXIN2, EDA, and EDAR genes [8, 10–15]. The genes responsible for nonsyndromic oligodontia are transforming growth factor-beta (TGF-*β*), PAX9, and MSX1 [8, 16–18].

Oligodontia (severe partial anodontia) is a developmental dental anomaly that refers to a congenital absence of more than six teeth, excluding third molars [19]. It has been previously reported in both primary and permanent teeth. It can be either associated with syndromes, a nonsyndromic isolated familial trait, or sporadic finding [5]. Gorlin et al. [20] reported that nonsyndromic oligodontia could be sporadic or familial.

Although reports on congenitally missing teeth are not new as these have been reported since ancient times (early reported from the Iron Age) [21], in the history of Saudi Arabia, significantly less data is available (Table 1) [22–29]. The prevalence of nonsyndromic congenitally missing primary teeth (0.24%) in Saudi Arabia has been reported by Yassin ^25^, which is lower compared to most countries (Table 2) [30–38]. Shilpa et al. [30] suggested that the possible causes for this variation are the difference in age groups included in these studies and geographical locations.

The reported case is unique for several reasons. Firstly, there are very few nonsyndromic nonfamilial cases reported with congenitally missing primary and permanent teeth. Secondly, this is the first nonfamilial and nonsyndromic oligodontia case reported in Saudi Arabia. Gholman et al. [39] reported a case of syndromic oligodontia, and Maganur et al. [40] reported oligodontia in permanent dentition. Thirdly, there are 21 missing teeth in the present case, which makes it an even rarer finding; previously, Venkataraghavan and Anantharaj [41] reported 18 missing teeth.

The diagnosis of oligodontia is usually based on routine clinical examination and radiographic evaluation while detecting missing teeth. Panoramic radiographs are preferred diagnostic aids as these register both the maxillary and mandibular arches and the developmental status of the permanent tooth buds with minimum radiation exposure [42]. For the present case, a diagnostic panoramic radiograph was avoided as the patient was extremely uncooperative, and only intraoral occlusal records were taken to reduce the radiation exposure (Figure 2).

Patients with oligodontia may experience significant psychological, esthetic, and functional issues. Therefore, it is essential to diagnose and treat these patients as soon as possible. The goal of the treatment includes saving the remaining teeth, recovering the masticatory and esthetic functions, improving speech, and restoring emotional and psychological well-being [42, 43]. Dental treatment is based on the severity of the disease and typically involves a multidisciplinary approach. The treatment choice depends on various factors like age of the patient, number and position of present teeth, patient's cooperation and treatment options, orthodontic therapy, implants, adhesive prosthesis, removable prosthesis, fixed prosthesis, and overdentures [42–44]. A treatment option is chosen based on functional and esthetic solutions. It allows the child as normal a lifestyle as possible without damaging or affecting the child's self-esteem, growth, and behavior [45]. In the present case, the treatment goal included behavior management, maintaining optimal oral health, educating about issues that may arise because of the long-term effects of both appliance therapy and spaced dentition, and monitoring of developing occlusion and eruption of permanent teeth. The treatment option chosen for the patient was a modified fixed Nance appliance with artificial upper anterior teeth.

Very few studies have measured oral health-related quality of life (OHRQoL) that have been carried out to provide some evidence that congenitally missing teeth may have an adverse impact on the quality of life [46]. Hobkirk et al. [47], Locker et al. [48], and Souza et al. [49] have, respectively, suggested that delayed treatment of such patients is likely to impact social and educational development negatively. In the present case, lack of socialization was reported due to missing teeth which was improved within a week after replacing missing teeth. Orthodontic therapy can be life-altering, and esthetically challenging cases such as this can help bring remarkable changes in a patient's esthetic profile [50, 51].

## 5. Conclusion

Oligodontia may be associated with a systemic condition and a syndrome or occur as an isolated event. Nonsyndromic oligodontia is a rare entity and usually remains undiagnosed till it affects esthetic or masticatory functions. The best way to detect both primary and permanent dentition congenital dental anomalies is through clinical and radiographical examination. A patient with missing teeth suffers not only from masticatory and esthetic problems but also from psychological stress, and it can lower the self-esteem of the individual. This case report illustrates the rare occurrence of oligodontia in both primary and permanent dentitions. An alternative treatment option treated the present case for a 4-year-old patient with agenesis of eight primary teeth. In addition to the effects of dental agenesis, in this situation, the child's mental and psychological health was a significant consideration for early intervention.

## Figures and Tables

**Table 1:** The prevalence of congenitally missing teeth in the different regions of Saudi Arabia.

S No.	Author name & year	Prevalence	Region
1	Tariq Alswayyed et al. (2018) [22]	33.7%	Riyadh
2	Tareq et al. (2017) [23]	7.42%	Aseer
3	Sajjad et al. (2016) [24]	6.1%	Al-Jouf
4	Yassin et al. (2016) [25]	9.7%	Abha
5	Vani et al. (2015) [26]	5.2%	Jazan
6	Al-Jabaa and Aldrees (2013) [27]	20%	—
7	Ghaznawi et al. (1999) [28]	9.41%	—
8	Salama and Abdel Megid (1994) [29]	2.6%	—

**Table 2:** The prevalence of nonsyndromic congenitally missing primary teeth worldwide.

S No.	Author name & year	Prevalence	Country
1	Shilpa et al. (2017) [30]	0.88%	South India
2	Mukhopadhyay and Mitra (2014) [31]	0.5%	East India
3	Kapdan et al. (2012) [32]	0.2%	Turkey
4	King et al. (2008) [33]	0.4-4.6%	Southern China
5	Kramer et al. (2008) [34]	0.6%	Brazil
6	Daugaard et al. (1997) [35]	54.9%	Denmark
7	Yonezu et al. (1997) [36]	2.38%	Japan
8	Whittington and Durward (1996) [37]	1.37%	New Zealand
9	Brook (1974) [38]	0.1-1.9%	Britain

## Data Availability

Any data related to the case report can be readily provided on reasonable request.
